# Influence of Resin Composition on the Photopolymerization of Zirconia Ceramics Fabricated by Digital Light Processing Additive Manufacturing

**DOI:** 10.3390/polym17101354

**Published:** 2025-05-15

**Authors:** Ning Kuang, Hao Qi, Wenjie Zhao, Junfei Wu

**Affiliations:** 1 College of Electromechanical Engineering, Qingdao University of Science and Technology, Qingdao 266061, China; kuang.ning@mails.qust.edu.cn (N.K.); 19558242420@163.com (H.Q.); 2 College of Sino-German Science and Technology, Qingdao University of Science and Technology, Qingdao 266061, China

**Keywords:** additive manufacturing, DLP, zirconia, photopolymerization

## Abstract

Digital light processing (DLP) is widely recognized as one of the most promising additive manufacturing technologies for ceramic fabrication. Nevertheless, during the additive manufacturing of zirconia ceramics, debinding and sintering often lead to structural defects, which severely deteriorate the material properties and hinder their broader application. In this study, we added an oligomer into the photosensitive resin and systematically investigated the effects of oligomer content on the viscosity and curing properties of ceramic suspensions. The results demonstrated that the introduction of oligomers is conducive to enhancing the crosslinking density and reducing defects. Finally, a 45 vol% solid content zirconia ceramic slurry was prepared by adding 20 wt% oligomers to the resin system. After printing, debinding, and sintering, the final zirconia ceramics exhibited a uniform microstructure without delamination or cracks, its bending strength reached 682.4 MPa. This study demonstrates that zirconia ceramics fabricated by photopolymerization with oligomer photosensitive resin exhibit excellent mechanical properties, significantly expanding the potential applications for high-performance zirconia ceramic components with additive manufacturing.

## 1. Introduction

Zirconia ceramics possess excellent properties, including high strength, toughness, wear resistance, and biocompatibility, which have facilitated their extensive use in both industrial and medical fields, such as in bearings, cutting tools, dental restorations, and bone implants [1,2,3]. Nevertheless, the significant hardness and inherent brittleness of zirconia ceramics create substantial difficulties when machining components with complex geometries using conventional manufacturing approaches. While methods like isostatic pressing, injection molding, and gel casting can be utilized to fabricate ceramics with moderately complex shapes [4], these techniques are highly dependent on molds and encounter several limitations, such as expensive mold production, complicated procedures, extended manufacturing times [5], and challenges in producing parts with highly intricate designs. These constraints have hindered the broader adoption of complex-shaped ceramic components.

Innovative rapid prototyping and manufacturing technologies have successfully addressed the inherent limitations of conventional fabrication methods, offering unprecedented flexibility in producing geometrically complex ceramic components [6]. Notably, additive manufacturing (AM), commonly referred to as 3D printing, is a layer-by-layer fabrication technology that enables direct digital-to-physical transformation [7]. This technology demonstrates multiple advantages, including rapid production rates, high forming accuracy, and low material costs [8,9]. Ceramic additive manufacturing primarily encompasses five key techniques: laminated object manufacturing (LOM), fused deposition modeling (FDM), selective laser sintering (SLS), direct ink writing (DIW), and photopolymerization-based methods. Among these, photopolymerization-based methods, including stereolithography (SL), liquid crystal display (LCD), and digital light processing (DLP), have emerged as the dominant approaches for fabricating ceramic components, owing to their superior dimensional accuracy and enhanced surface quality [10,11,12].

**Figure 1:**
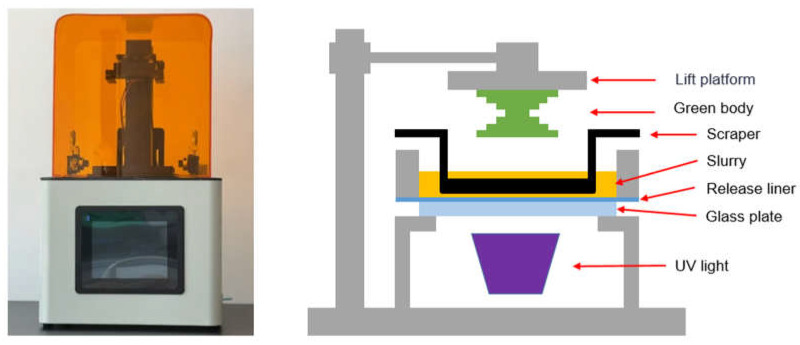
DLP printer and schematic diagram.

The working principle of DLP technology is to project images with high-resolution digital projectors for layer-by-layer curing. These images are then stacked to form objects, as shown in Figure 1. Since each layer is formed in a single exposure, DLP features high precision, high speed, and high cost-effectiveness [13]; therefore, it is more widely applied in ceramic additive manufacturing. DLP technology operates through a photochemical mechanism in which UV irradiation triggers the crosslinking of photosensitive resin components, resulting in controlled polymerization and layer-by-layer solidification, while simultaneously curing the resin-coated ceramic powder [14,15].

Given the characteristics of the photopolymerization process, the ceramic slurry must possess excellent fluidity, low viscosity, and outstanding photopolymerization performance. Additionally, to minimize defects such as cracks and achieve a dense ceramic body, the ceramic slurry must also have a high solid loading [16,17,18]. Typically, the photosensitive resin in the slurry comprises various monomers to lower viscosity and enhance the curing character for effective photocuring reactions [19,20]. Therefore, selecting an appropriate photosensitive resin formulation is crucial for ensuring slurry stability while simultaneously meeting the requirements of both high solid loading and low viscosity.

In our previous studies, a photosensitive resin formulation with relatively low viscosity and a high photopolymerization performance was successfully prepared [21]. However, previous studies have predominantly focused on monomer screening in photosensitive resin systems, overlooking the fact that small-molecule monomers exhibit significant contraction rates, which can lead to cracks and deformations in ceramic components [22]. Recent research has shown several advancements. Xu et al. [23] successfully optimized resin–ceramic adhesion by precisely controlling double bond density and minimizing curing shrinkage. Johansson et al. [24] added non-reactive components 2-[[2-(Benzoyloxy)ethyl]amino]ethanol (BEA) and polyethylene glycol (PEG-200) to the photosensitive resin, which helped reduce polymerization shrinkage and modified the thermal decomposition of the polymer matrix. This optimization effectively suppressed layer delamination and interlayer cracking during thermal processing, yielding sintered components with a near-theoretical relative density of 99%. In a parallel study, Dang et al. [4] strategically replaced a portion of the ethoxylated pentaerythritol tetraacrylate (PPTTA) monomer with ethoxylated trimethylolpropane triacrylate (ETPTA) and incorporated functional oligomers. This formulation redesign reduced the critical exposure energy to 14.88 mJ/cm^2^ while achieving a 98.5% monomer conversion rate. Han et al. [5] studied the influence of different PEG-200 contents on the degreasing process and mechanical properties of zirconia parts, and it was proved that PEG-200 could effectively inhibit the defects during the degreasing process.

From these studies, it is clear that reducing the effects of shrinkage helps prevent cracks and structural defects, ultimately improving the mechanical and functional performance of ceramics. Oligomer is a component of partial photosensitive resins that, compared to monomers, polymerizes with far fewer double bonds being consumed. As a result, the photopolymerization of oligomers induces substantially less volume shrinkage [22]. However, there are relatively few studies on the influence of oligomers on the photosensitive resin system of zirconia ceramics.

In this study, based on our previous research on resins [21], oligomers were introduced into the photosensitive resin system, and the effects of different contents of oligomers on the viscosity and photopolymerization performance of zirconia ceramic slurry were investigated. Through a comprehensive investigation of microstructure evolution and its impact on mechanical properties, the optimal balance point for the monomer-to-oligomer ratio in the photosensitive resin system was successfully identified. The zirconia ceramic slurry prepared with the resin containing a definite amount of oligomer was subjected to printing, debinding, sintering, and testing, resulting in significant improvements in the properties of the ceramic finish part.

## 2. Materials and Methods

### 2.1. Materials

In this study, zirconia powder (D50 = 1 µm, Shandong Sitaili Metal Materials Co., Ltd., Jinan, China) was used. The resins used in this study, including monomers Acryloyl morpholine (ACMO, Shanghai Guangyi Chemical Co., Ltd., Shanghai, China), 1,6-Hexanediol diacrylate (HDDA, Shanghai Guangyi Chemical Co., Ltd., Shanghai, China), Trimethylolpropane triacrylate (TMPTA, Shanghai Guangyi Chemical Co., Ltd., Shanghai, China), and oligomer Bisphenol A epoxy acrylate (BAEA, Shanghai Yinchang New Materials Co., Ltd., Shanghai, China). A 2,4,6-trimethyl benzoyl diphenyl phosphine oxide (TPO, Shanghai Guangyi Chemical Co., Ltd., Shanghai, China) was used as the photoinitiator, while BYK110 (Dongguan Haoyouduo New Materials Co., Ltd., Dongguan, China) was selected as a dispersant.

### 2.2. Preparation of Zirconia Ceramic Slurry

**Figure 2:**
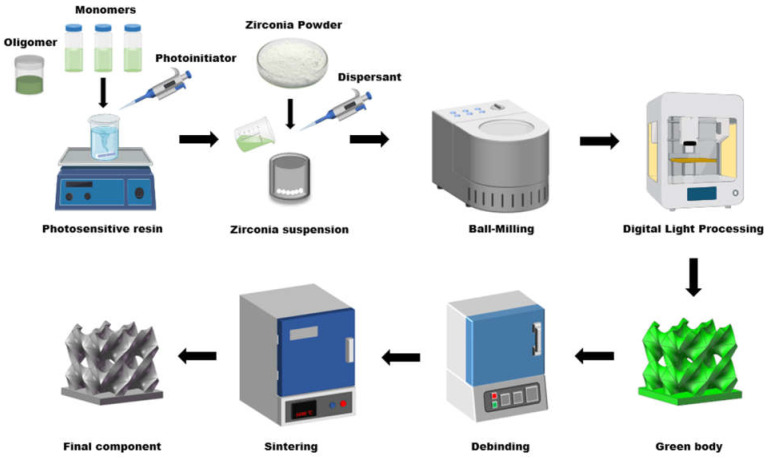
3D printing zirconia ceramic fabrication process.

As shown in Figure 2, first, the photosensitive resin was prepared by mixing different resin monomers and oligomers via a magnetic stirrer (RCT-Basic, IKA-Werke GmbH & Co. KG, Staufen im Breisgau, Germany) at 500 r/min for 60 min. The monomer and oligomer compositions of the different resin groups are shown in Table 1. Next, a specified amount of zirconia ceramic powder was added to the prepared photosensitive resin, different content of dispersant was added to the suspension, ranging from 1 wt% to 3 wt% of zirconia powder, and TPO with 3 wt% of resins was added as the photoinitiator. The suspension was then ball-milled using a planetary ball mill (BQM-1L, Changsha Miqi Instruments & Equipment Co., Ltd., Changsha, China) at 350 rpm, with alternating forward and reverse rotations for 10 h each, resulting in a homogenously dispersed zirconia ceramic slurry.

### 2.3. Fabrication

The zirconia ceramic green body was printed using a DLP ceramic printer (BLD-25-C1, Qingdao Breuck 3D Additive Manufacturing Co., Ltd., Qingdao, China). The print thickness was selected as 30 µm, with the exposure power between 3 and 6 mW/cm^2^, and an exposure time of 3–30 s. The green body was fabricated in a rectangular geometry measuring 35 mm × 4 mm × 3 mm. After printing, the green body was treated through debinding according to the thermogravimetric analysis. The zirconia green body was debound at 600 °C in air atmosphere in debinding furnace (FMJ-07/11, Hefei Facerom Intelligent Equipment Co., Ltd., Hefei, China) for 2 h and then sintered in the sintering furnace (FMJ-05/17, Hefei Facerom Intelligent Equipment Co., Ltd., Hefei, China) with a heating rate of 5 °C/min to 1600 °C for 2 h.

### 2.4. Characterization

**Figure 3:**
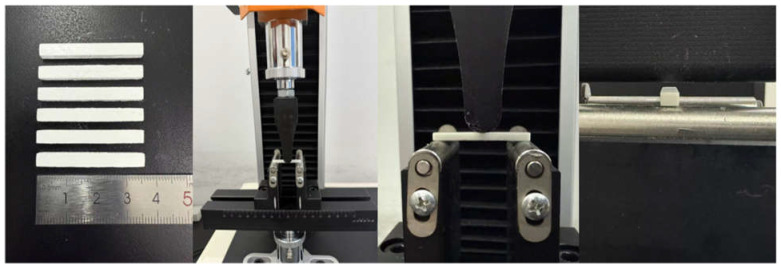
Three-point bending test.

The rheological property of zirconia slurries was determined using a digital viscometer (NDJ-8Spro, Shanghai Xiniulab Instruments Co., Ltd., Shanghai, China) at room temperature. The curing thickness was measured using a micrometer (Q2LF0025, Deqing Shengtaixin Electronic Technology Co., Ltd., Deqing, China). The thermal decomposition characteristics of the green body were investigated using thermogravimetric analysis (TGA; Labsys EVO, SETARAM Instrumentation, Caluire et Cuire, France) with a constant heating rate of 5 °C/min under a flowing air atmosphere. A scanning electron microscope (SEM, SU8010, HITACHI, Tokyo, Japan) characterization was performed to analyze the microstructural transformations from the green body through debinding to the sintering state. The sintered ceramic specimens were tested for three-point bending strength using a testing machine (Digital electronic universal testing machine, WH-70, Ningbo Weiheng Testing Instruments Co., Ltd., Ningbo, China), as shown in Figure 3, at a loading rate of 0.5 mm/min. To ensure repeatability, five samples were tested.

## 3. Results and Discussion

### 3.1. Rheological Properties of Zirconia Ceramic Slurry

**Figure 4:**
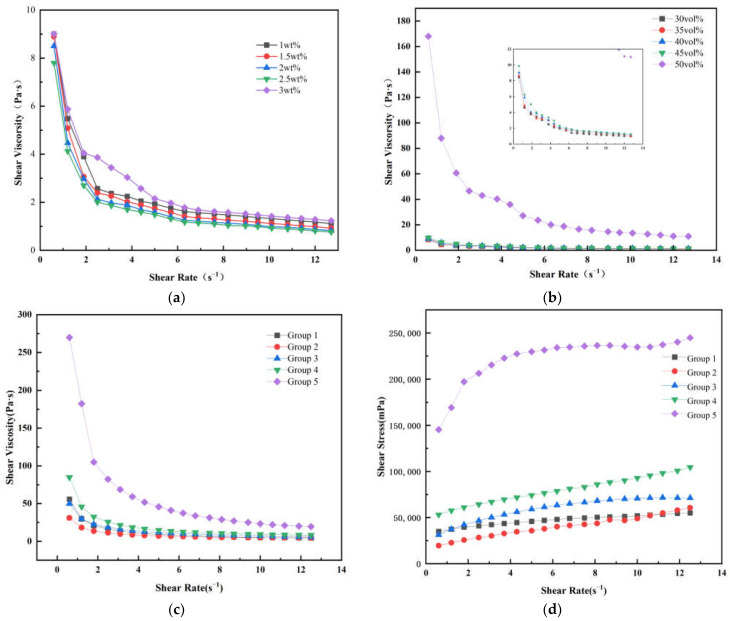
(**a**) Relationship between shear viscosity and shear rate of zirconia ceramic slurry with different dispersant content. (**b**) Rheological properties of zirconia ceramic slurry with different solid loading. (**c**) Relationship between shear viscosity and shear rate with different resin components. (**d**) Relationship between shear stress and shear rate with different resin components.

In order to prepare a slurry of zirconia ceramics with low viscosity and good dispersion properties, a dispersant was added to the slurry. Determining the optimal dispersant concentration represents a crucial parameter for attaining target rheological properties in high solid-loading slurries for ceramic additive manufacturing applications. To determine the optimal concentration of dispersant for the zirconia slurry, the rheological properties of suspensions containing varying dispersant concentrations (1, 1.5, 2.0, 2.5, and 3 wt% relative to zirconia powder) were investigated, as shown in Figure 4a; for the convenience of comparison, a slurry with resin Group 1 and 40 vol% solid content was used. It has been observed that the viscosity of all the slurries with different contents of dispersant decreases as the shear rate increases, and all the slurries exhibit non-Newtonian fluid characteristics. The viscosity decreases as the content of the dispersant increases from 1% to 2.5 wt%, but starts to increase when the content further increases to 3 wt%. This phenomenon is commonly ascribed to molecular flocculation induced by excessive dispersant dosage, resulting in impaired suspension fluidity [25,26]. Based on the rheological characterization results, a dispersant concentration of 2.5 wt% was determined to be optimal and was therefore selected for all subsequent processing steps in this investigation.

The solid loading of the ceramic suspension was further optimized by assessing its rheological behavior, as shown in Figure 4b. As the solid content increases, the slurry viscosity rises accordingly. At 50 vol% solid loading, a sharp increase in viscosity is observed. A good slurry fluidity is essential for smooth printing; normally, the viscosity should be less than 10 Pa·s at a shear rate of 10 s^−1^ [27]. When subjected to a shear rate of 10 s^−1^, the slurry containing 50% solids demonstrates a viscosity of 20 Pa·s, and such a high viscosity is no longer suitable for printing. Therefore, the final solid loading of the zirconia ceramic slurry was set at 45 vol% to ensure the printing process.

Based on the dispersant and solid loading research, the influence of oligomer content on rheological behavior has been studied. As shown in Figure 4c, the viscosity of the slurry progressively increases with increasing oligomer concentration. This occurs because the oligomers exhibit higher viscosity compared to photosensitive resin monomers, thereby increasing the overall slurry viscosity. The viscosities at a typical shear rate of 10 s^−1^ were 5.18, 4.90, 7.06, and 9.25 Pa·s for the slurries of Groups 1, 2, 3, and 4, respectively, demonstrating that these slurries are suitable for the printing process. In contrast, Group 5 exhibited a viscosity of 23.36 Pa·s at a 10 s^−1^ shear rate, exceeding the printable range.

As shown in Figure 4d, the shear stress also increases with the increase in oligomer content. The shear stresses at a typical shear rate of 10 s^−1^ were 51,941, 48,994, 70,851, and 92,830 mPa for the slurries of Groups 1, 2, 3, and 4, respectively. Under the same conditions, the shear stress of the Group 5 was 234,655 mPa, which is significantly higher than that of the other groups. This indicates that when oligomers completely replace multifunctional monomers, the rheological properties of the slurry deteriorate significantly.

Due to the inadequate rheological properties of the slurry containing resin Group 5, it is no longer possible to complete the printing; therefore, this study subsequently utilized only slurries with resin Groups 1, 2, 3, and 4 for all further testing.

### 3.2. Photocuring Performance of Zirconia Ceramic Slurry

During light exposure, the photoinitiator in the photosensitive resin system generates free radicals that can initiate the polymerization of monomers or oligomers. This leads to the polymerization and crosslinking of active monomers or oligomers within the photosensitive materials, ultimately forming a three-dimensional network structure.

In the presence of a photoinitiator, the photosensitive resin premix undergoes photopolymerization under specific light intensity, leading to the formation of a crosslinked network structure. The cure depth C_d_ (μm) of the resin is influenced by the energy dose and can be described using the Beer–Lambert law [28], which relates light attenuation to material properties. According to this model, the energy imparted to the resin diminishes with increasing depth, resulting in a decrease in cure depth as the layer thickness increases. The relationship between cure depth and energy dose can be expressed as follows:

(1) $$C_{d}=D_{p}\ln\left(\frac{E}{E_{c}}\right)$$

**Figure 5:**
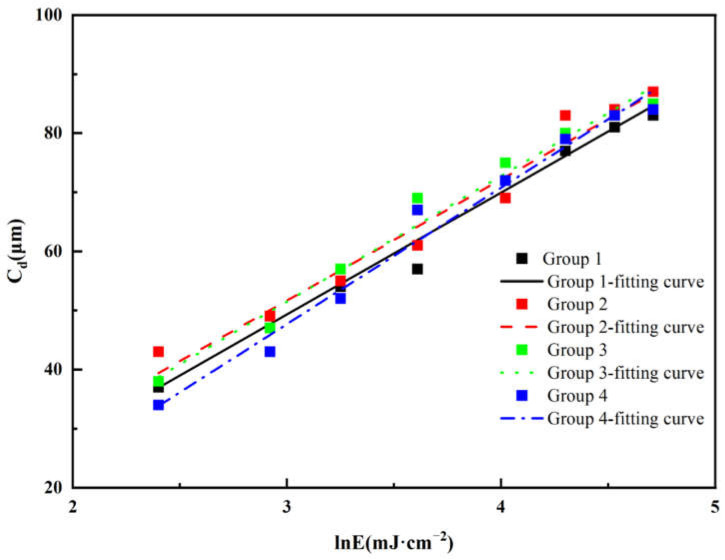
Linear fitting relationship between exposure energy and cure depth of the zirconia ceramic slurries with different resin compositions.

Here, projection depth D_p_ (μm) represents the penetration depth of the experimental setup, E_c_ (mJ·cm^−2^) is the critical exposure energy, and E is the actual exposure energy (mJ·cm^−2^) applied. This equation indicates that the cure depth increases logarithmically with the energy dose. In this study, we analyzed the relationship between C_d_ and lnE, as illustrated in Figure 5, and performed curve fitting to determine the variations in D_p_ and E_c_, along with their corresponding R^2^ values, as shown in Table 2, to assess the model’s accuracy.

All experimental curing depths exceeded the 30 μm layer thickness, fulfilling the fundamental printing specifications. As evidenced by the fitted data, both the projection depth (D_p_) and critical exposure energy (E_c_) exhibited a decreasing trend with increasing oligomer content. Notably, Group 4 displayed the highest values among all ceramic slurries, suggesting superior photosensitivity that enables curing at lower energy levels compared to Groups 1–3. Furthermore, the high correlation coefficients (R^2^) observed across all groups indicate excellent fitting reliability.

### 3.3. TG-DTG Analysis

**Figure 6:**
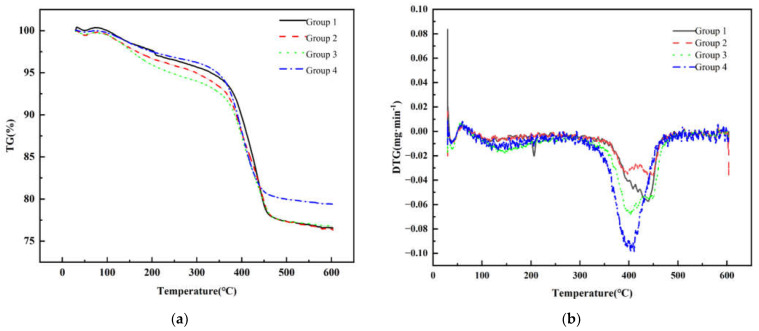
(**a**) TG curves of printed zirconia ceramic green body with different resin compositions. (**b**) DTG curves of printed zirconia ceramic green body with different resin compositions.

As evidenced by the TG-DTG results in Figure 6, the primary decomposition temperature ranges of organic components occurred between 400 °C and 450 °C. Near-complete thermal decomposition was achieved by 600 °C, after which the green body mass stabilized. Consequently, during the debinding process, a slow heating rate is maintained up to 100 °C to ensure thorough moisture removal without inducing cracks. Furthermore, an isothermal holding stage was implemented around 400 °C and 450 °C. The epoxy acrylate cured by photopolymerization has enhanced thermal stability due to its crosslinked network structure. The decomposition initiation temperature has risen to 300–350 °C, and the main decomposition stage is concentrated in the range of 350–450 °C [29]. The test results also verified this conclusion, with the increase in oligomer content, the resin decomposition process becomes more violent. As shown in Figure 6b, the debinding processes in Groups 1, 2, and 3 are much milder, among them, Group 3 exhibits the widest decomposition temperature range and shows a distinct multi-stage decomposition process, thereby effectively minimizing the formation of internal defects.

### 3.4. Properties of Zirconia Ceramics

**Figure 7:**
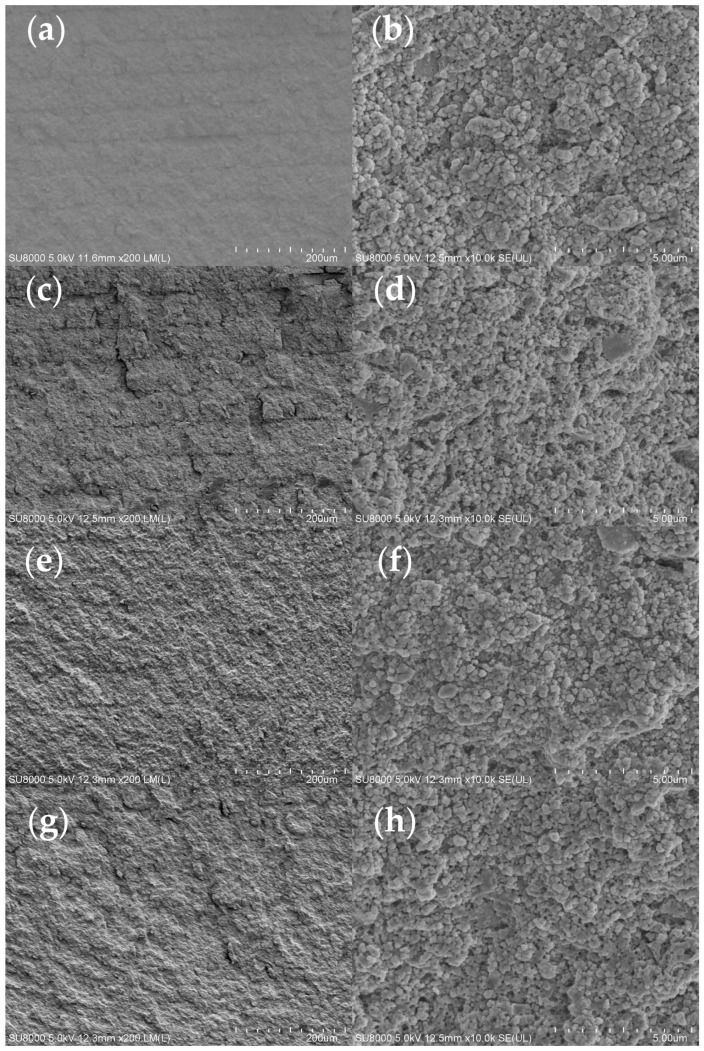
SEM images of green body fracture surfaces with different resin groups: (**a**,**b**) Group 1; (**c**,**d**) Group 2; (**e**,**f**) Group 3; (**g**,**h**) Group 4.

Ceramic specimens were, respectively, printed using slurries with four different group resins. The SEM micrographs of the printed green body are shown in Figure 7. As can be seen from the figures of the green body with Group 1 and Group 2, the interlayer cracks between each layer can be clearly seen. This is likely caused by the low polymerization degree of the polymers in the slurry. In contrast, the green body of Groups 3 and 4 exhibited nearly indistinguishable interlayer cracks from the printing process; these results demonstrate that the addition of oligomers was found to promote more complete photopolymerization and increase crosslinking density.

**Figure 8:**
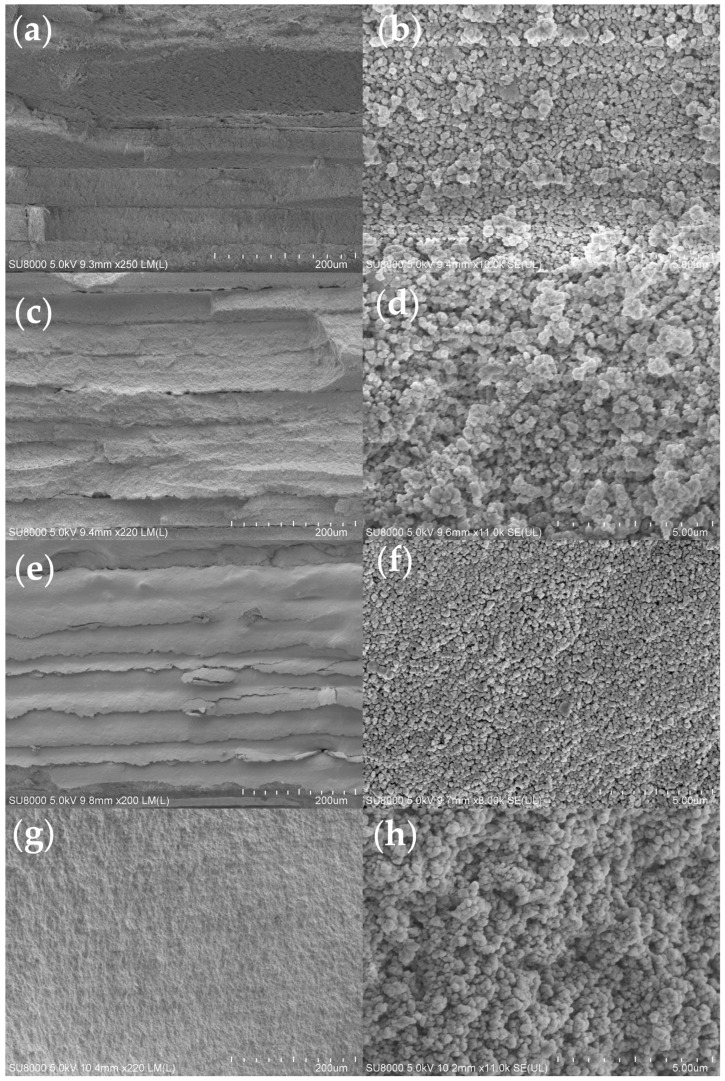
SEM images of debinded ceramic specimens with different resin groups: (**a**,**b**) Group 1; (**c**,**d**) Group 2; (**e**,**f**) Group 3; (**g**,**h**) Group 4.

Figure 8 presents SEM images comparing the surface morphologies of debinded samples fabricated by different group resin composites. During the debinding process, polymer decomposition leads to significant volumetric shrinkage. The micrographs in Figure 8a,c,e clearly reveal interfacial delamination between layers, demonstrating insufficient interlayer bonding strength. However, no delamination is observed in Figure 8g, demonstrating that the oligomer addition effectively mitigates interlayer cracks and, consequently, enhances the mechanical properties of the components. Additionally, it can be observed from Figure 8f that the ceramic particles are distributed very uniformly, with no obvious defects detected. This is attributed to the relatively smooth debinding process of Group 3, which also aligns with the TG-DTG results.

**Figure 9:**
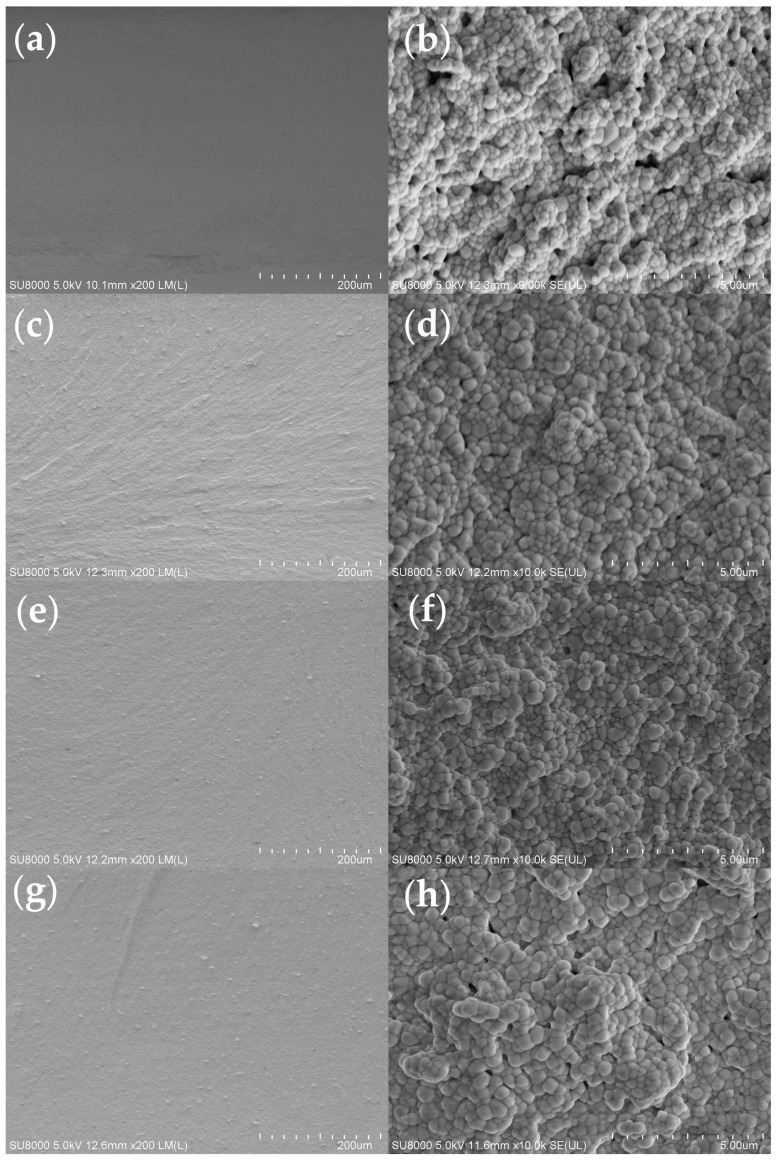
SEM images of the bending fracture surfaces of the sintered specimens with different resin groups: (**a**,**b**) Group 1; (**c**,**d**) Group 2; (**e**,**f**) Group 3; (**g**,**h**) Group 4.

**Figure 10:**
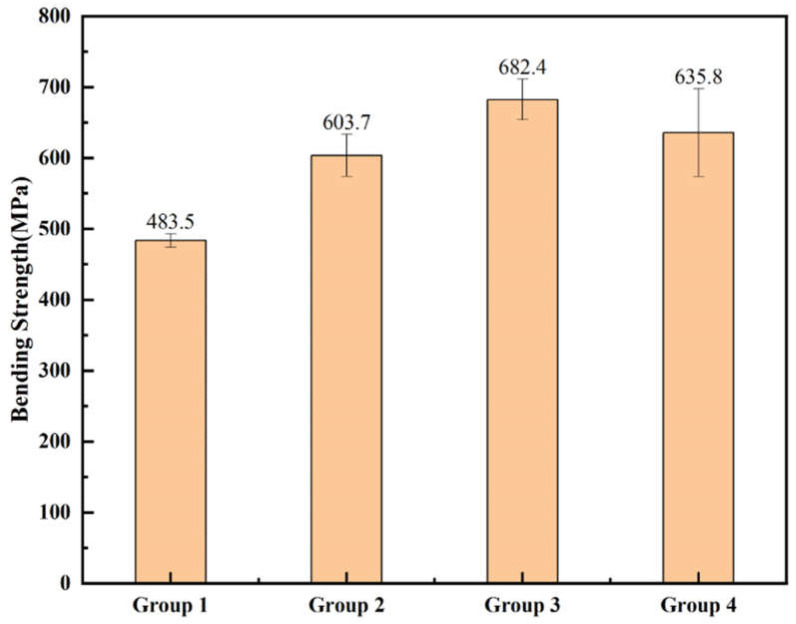
Bending strength of sintered zirconia ceramic specimens with different resin groups.

Figure 9 presents the fracture surface morphologies of sintered ceramics specimens with different resin groups. It could be clearly observed that in samples of Groups 2, 3, and 4, interlayer cracks almost disappeared. Furthermore, higher-magnification microstructural examination demonstrates that the specimen with Group 3 resin has the least amount of micropores remaining inside the ceramics. The three-point bending test also confirmed this result. As shown in Figure 10, the bending test of specimens with oligomer (Groups 2, 3, and 4) exhibited significantly enhanced performance compared to Group 1, in which no oligomer was added. Among the tested groups, Group 3 demonstrated in all the tested resin groups the highest bending strength of 682.4 MPa.

These results further indicate that the resin formulation explored in this study is suitable for DLP additive manufacturing of zirconia ceramics, and they are helpful in reducing defects that occur during the printing, debinding, and sintering processes, and enhancing the mechanical properties of zirconia ceramics.

## 4. Conclusions

In this study, the effects of different oligomer content on the rheological properties, the photopolymerization behavior of slurries, and the mechanical properties of printed ceramic specimens were systematically investigated, along with the influence of dispersant content and solid loading on the rheological properties of the zirconia ceramic slurry.

1. Dispersant Content Impact: This study elucidated the critical role of dispersants in controlling rheological behavior, with the 2.5 wt% dispersant formulation showing minimum viscosity among all tested compositions.
2. Solid loading Impact: The addition of a 2.5 wt% dispersant enabled the achievement of an optimal slurry solid loading of 45 vol%.
3. Oligomer Content Impact: This study revealed that although the addition of oligomers negatively affects the rheological properties of the slurry, it enhances the photopolymerization performance, increases the degree of polymerization and crosslinking density in DLP printing, and helps reduce interlayer cracks, thereby improving the strength of printed parts. The zirconia ceramic samples fabricated using a slurry containing 20 wt% oligomers of photosensitive resin exhibited a bending strength of 682.4 MPa after printing, debinding, and sintering processes.

## Figures and Tables

**Table 1:** Composition of resin.

Group	ACMO (wt%)	HDDA (wt%)	TMPTA (wt%)	BAEA (wt%)
1	50	10	40	0
2	50	10	30	10
3	50	10	20	20
4	50	10	10	30
5	50	10	0	40

**Table 2:** Photocuring characteristic parameters of zirconia ceramic slurry with different resin compositions.

Group	D_p_	E_c_	R^2^
1	20.64	1.840	0.97095
2	20.48	1.611	0.96357
3	21.26	1.783	0.97718
4	23.09	2.546	0.97713

## Data Availability

The original contributions presented in this study are included in the article. Further inquiries can be directed to the corresponding authors.
